# Supplementary material for: Deletion of wfs1 Impairs Oligodendrocyte Precursor Cells Dorsal Distribution and Myelination Through the wfs1-hmgcs1 Axis in Zebrafish
Source: Biology (Basel). 2026 Aug 16;15(16):1402. doi: 10.3390/biology15161402 (PMC13510151; doi:10.3390/biology15161402)
Supplement: Supplementary file 1 [file biology-15-01402-s001.zip › File S1-Transcriptome sequencing data and differentially expressed genes (DEGs) in wfs1-- zebrafish at 5 dpf/1.GO/wfs1KOvsWT/GSEA/WT/HABENULA_DEVELOPMENT/HABENULA_DEVELOPMENT(GO_0021986).html]

Details for gene set HABENULA\_DEVELOPMENT(GO:0021986)[GSEA]


|  || Dataset | deg.sample |
| Phenotype | sample.cls |
| Upregulated in class | WT |
| GeneSet | HABENULA\_DEVELOPMENT(GO:0021986) |
| Enrichment Score (ES) | -0.53363794 |
| Normalized Enrichment Score (NES) | -1.6957648 |
| Nominal p-value | 0.0 |
| FDR q-value | 0.32041523 |
| FWER p-Value | 0.927 |
Table: GSEA Results Summary

  

Fig 1: Enrichment plot: HABENULA\_DEVELOPMENT(GO:0021986)
  
Profile of the Running ES Score & Positions of GeneSet Members on the Rank Ordered List

  

| PROBE | DESCRIPTION   (from dataset) | GENE SYMBOL | GENE\_TITLE | RANK IN GENE LIST | RANK METRIC SCORE | RUNNING ES | CORE ENRICHMENT || 1 | 373865 | kctd12.1 |  |  | 4493 | 0.746 | -0.0729 | No |
| 2 | 566632 | med12 |  |  | 7723 | 0.440 | -0.1340 | No |
| 3 | 553403 | kctd12.2 |  |  | 7799 | 0.437 | -0.0964 | No |
| 4 | 30269 | shha |  |  | 8146 | 0.415 | -0.0694 | No |
| 5 | 100329702 | ulk2 |  |  | 9563 | 0.329 | -0.0838 | No |
| 6 | 30239 | neurog1 |  |  | 12014 | 0.190 | -0.1432 | No |
| 7 | 192298 | sec61a1 |  |  | 13031 | 0.138 | -0.1625 | No |
| 8 | 352910 | mib1 |  |  | 14113 | 0.087 | -0.1885 | No |
| 9 | 557451 | daam1a |  |  | 14389 | 0.075 | -0.1903 | No |
| 10 | 30225 | smo |  |  | 15602 | 0.017 | -0.2267 | No |
| 11 | 30718 | notch1a |  |  | 15928 | 0.001 | -0.2368 | No |
| 12 | 406420 | wls |  |  | 15947 | 0.000 | -0.2373 | No |
| 13 | 30567 | pax6a |  |  | 16632 | -0.021 | -0.2569 | No |
| 14 | 30510 | tcf7l2 |  |  | 17360 | -0.048 | -0.2753 | No |
| 15 | 30476 | wif1 |  |  | 25579 | -0.492 | -0.4882 | Yes |
| 16 | 30164 | pitx2 |  |  | 27028 | -0.632 | -0.4760 | Yes |
| 17 | 30123 | wnt4a |  |  | 27492 | -0.672 | -0.4291 | Yes |
| 18 | 368439 | mycbp2 |  |  | 30473 | -1.490 | -0.3866 | Yes |
| 19 | 266958 | neurod4 |  |  | 31812 | -4.720 | 0.0024 | Yes |
Table: GSEA details
[plain text format]

  

Fig 2: HABENULA\_DEVELOPMENT(GO:0021986)
  
Blue-Pink O' Gram in the Space of the Analyzed GeneSet

  

Fig 3: HABENULA\_DEVELOPMENT(GO:0021986): Random ES distribution
  
Gene set null distribution of ES for
 **HABENULA\_DEVELOPMENT(GO:0021986)**
